# Expression and Localization of Cathepsins B, D, and G in Two Cancer Stem Cell Subpopulations in Moderately Differentiated Oral Tongue Squamous Cell Carcinoma

**DOI:** 10.3389/fmed.2017.00100

**Published:** 2017-07-20

**Authors:** Therese Featherston, Reginald Walter Marsh, Bede van Schaijik, Helen D. Brasch, Swee T. Tan, Tinte Itinteang

**Affiliations:** ^1^Gillies McIndoe Research Institute, Wellington, New Zealand; ^2^University of Auckland, Auckland, New Zealand; ^3^Wellington Regional Plastic, Maxillofacial and Burns Unit, Hutt Hospital, Wellington, New Zealand

**Keywords:** oral tongue, squamous cell carcinoma, cancer, cathepsin, renin–angiotensin system, cancer stem cells, oral cavity, head and neck

## Abstract

**Aim:**

We have previously demonstrated the putative presence of two cancer stem cell (CSC) subpopulations within moderately differentiated oral tongue squamous cell carcinoma (MDOTSCC), which express components of the renin–angiotensin system (RAS). In this study, we investigated the expression and localization of cathepsins B, D, and G in relation to these CSC subpopulations within MDOTSCC.

**Methods:**

3,3-Diaminobenzidine (DAB) and immunofluorescent (IF) immunohistochemical (IHC) staining was performed on MDOTSCC samples to determine the expression and localization of cathepsins B, D, and G in relation to the CSC subpopulations. NanoString mRNA analysis and colorimetric *in situ* hybridization (CISH) were used to study their transcripts expression. Enzyme activity assays were performed to determine the activity of these cathepsins in MDOTSCC.

**Results:**

IHC staining demonstrated expression of cathepsins B, D, and G in MDOTSCC. Cathepsins B and D were localized to CSCs within the tumor nests, while cathepsin B was localized to the CSCs within the peri-tumoral stroma, and cathepsin G was localized to the tryptase^+^ phenotypic mast cells within the peri-tumoral stroma. NanoString and CISH mRNA analyses confirmed transcription activation of cathepsins B, D, and G. Enzyme activity assays confirmed active cathepsins B and D, but not cathepsin G.

**Conclusion:**

The presence of cathepsins B and D on the CSCs and cathspsin G on the phenotypic mast cells suggest the presence of bypass loops for the RAS which may be a potential novel therapeutic target for MDOTSCC.

## Introduction

Oral cavity cancers are the eighth most common cancer worldwide (1) accounting for 2% of cancer mortality (2). Oral tongue squamous cell carcinoma (OTSCC) is the most common oral cavity cancer (3). Risk factors for OTSCC include tobacco and alcohol consumption which act synergistically to promote carcinogenesis (1).

Current mainstay treatment for OTSCC is surgery often with post-operative radiotherapy, and sometimes chemotherapy (4). The 5-year survival rate of 50–55% for OTSCC (3) has remained largely unchanged over the past 40 years (5).

Cancer stem cells (CSCs), demonstrated in many types of cancers, have been proposed to be the origin of cancer including OTSCC (6). Increased tumor size, local invasion, local recurrence, and regional metastasis have been associated with overexpression of CSC markers (7).

We have recently characterized two CSC subpopulations within moderately differentiated oral tongue squamous cell carcinoma (MDOTSCC) with an OCT4^−^ subpopulation within the tumor nests (TNs) that also expresses EMA; and an OCT4^+^ subpopulation within the peri-tumoral stroma that does not express EMA (6).

The renin–angiotensin system (RAS) is a hormonal system classically associated with blood pressure and body fluid regulation. Recent literature has demonstrated its role in cancer growth and metastasis (8) by promoting angiogenesis and cell proliferation (9, 10). Angiotensinogen (ANG) undergoes conversion to angiotensin I (ATI) by renin, the active form of pro-renin (8, 11). Pro-renin receptor (PRR) is the receptor for both pro-renin and renin (12). ATI is then converted to angiotensin II (ATII) by the action of angiotensin converting enzyme (ACE) (8). Vasoactive ATII acts on angiotensin II receptor 1 (ATIIR1) and angiotensin II receptor 2 (ATIIR2) (8).

We have recently demonstrated the presence of two putative CSC subpopulations in MDOTSCC: one within the TNs and the other within the peri-tumoral stroma (6). We have also demonstrated the expression of components of the RAS by these CSCs (13). The CSC subpopulation within the TNs expresses PRR, ATIIR1, and ATIIR2, while the CSC subpopulation within the peri-tumoral stroma expresses PRR, ACE, ATIIR1, and ATIIR2 (13).

Cathepsins B, D, and G are proteases that provide putative bypass loops for the RAS. Cathepsin B, a cysteine protease, is a pro-renin processing enzyme and converts inactive pro-renin to active renin. Cathepsin D, an aspartyl protease, is functionally homologous to renin. Cathepsin G, a serine protease, can directly produce ATII from ANG and ATI, and is functionally homologous to ACE (14). We have previously demonstrated an ESC-like population within infantile hemangioma (15) that expresses components of the RAS (14, 15) and also cathepsins B, D, and G, suggesting the existence of bypass loops (14).

In this study, we investigated the expression and localization of cathepsins B, D, and G in relation to the CSC subpopulations within MDOTSCC. NanoString mRNA analysis and colorimetric *in situ* hybridization (CISH) were used to confirm mRNA transcription. Protein expression and localization of each cathepsin was determined by 3,3-diaminobenzidine (DAB) and immunofluorescent (IF) immunohistochemical (IHC) staining. Enzymatic activity assays were performed to demonstrate the activity of these cathepsins.

## Materials and Methods

### Tissue Samples

Moderately differentiated oral tongue squamous cell carcinoma samples from four male and five female patients, aged 30–73 (mean, 58.2) years, were sourced from the Gillies McIndoe Research Institute Tissue Bank for this study, which was approved by the Central Regional Health and Disability Ethics Committee (ref. no 12/CEN/74). Written consent was obtained from all participants.

### Histochemical and IHC Staining

Hematoxylin and eosin (H&E) staining was used to confirm the presence and appropriate histological grading on 4 μm thick formalin-fixed paraffin-embedded of MDOTSCC sections from nine patients by an anatomical pathologist (HDB). These MDOTSCC sections were then used for DAB IHC staining, as previously described (6, 13), using primary antibodies for cathepsin B (1:1,000; cat# sc-6490-R, Santa Cruz, CA, USA), cathepsin D (1:200; cat# NCL-CDm, Leica, Newcastle upon Tyne, UK), cathepsin G (1:200; cat# sc-33206, Santa Cruz, CA, USA), EMA (ready-to-use, cat# PA0035, Leica), OCT4 (1:30, cat# MRQ-10, Cell Marque, Rocklin, CA, USA), and tryptase (1:300, cat# NCL-MCTRYP-428, Leica). All DAB IHC-stained slides were mounted in Surgipath Micromount (Leica).

Immunofluorescent (IF) IHC staining was performed to determine co-expression of two proteins on two samples of MDOTSCC from the original cohort of nine patients used for DAB IHC staining. Vectafluor Excel anti-mouse 488 (ready-to-use; cat# VEDK2488, Vector Laboratories, Burlingame, CA, USA) and Alexa Fluor anti-rabbit 594 (1:500; cat# A21207, Life Technologies, Carlsbad, CA, USA) were utilized to detect the combinations. All IF IHC-stained slides were mounted in Vecta Shield Hardset mounting medium with 4′,6′-diamino-2-phenylindole (Vector Laboratories).

Positive control tissues used for the primary antibodies were human placenta for cathepsin B; human breast cancer for cathepsin D; mouse bone marrow for cathepsin G; and human seminoma for OCT4 and SALL4. A negative MDOTSCC control sample was prepared for DAB IHC staining by using an IgG isotype control (ready-to-use; cat# IR600, Dako, Santa Clara, CA, USA). For IF IHC staining, a negative control was performed using a section of MDOTSCC tissue with the combined use of primary isotype mouse (ready-to-use; cat# IR750, Dako, Copenhagen, Denmark) and rabbit (read-to-use; cat# IR600, Dako) antibodies.

All antibodies were diluted with Bond primary antibody diluent (cat# AR9352, Leica), and DAB and IF IHC staining was carried out on the Leica Bond Rx autostainer, as previously described (16).

### NanoString mRNA Analysis

Six snap-frozen samples of MDOTSCC from the original cohort of nine patients used for DAB IHC staining were used for isolation of total mRNA for NanoString nCounter™ Gene Expression Assay (Nanostring Technologies, Seattle, WA, USA), as previously described (6, 13). Probes for the genes encoding for cathepsin B (NM_001908.2), cathepsin D (NM_001909.3), cathepsin G (NM_001911.2), and the housekeeping gene PGK1 (NM_000291.3) were used in the analysis. Raw data were analyzed by nSolver™ software (NanoString Technologies). Results were normalized against the housekeeping gene, graphed using Excel (Microsoft Office 2013), and subjected to *t*-tests for related samples, to compare the relative abundance of each cathepsin.

### Colorimetric *In Situ* Hybridization

4 μm thick formalin-fixed paraffin-embedded sections of six samples of MDOTSCC from the original cohort of nine patients used for DAB IHC staining were used for CISH. Staining was carried out on the Leica Bond Rx auto-stainer and detected using the ViewRNA red stain kit (Affymetrix, Santa Clara, CA, USA), as previously described (16). The probes used for cathepsin B (cat# VA1-12282), cathepsin D (cat# VA1-12281), cathepsin G (cat# VA1-21016), and *Bacillus* (cat# VF1-11712, Affymetrix) as a negative control. Positive controls used were the same for DAB IHC staining.

### Cell Counting and Statistical Analyses

Cell counting was performed on six fields of view of the DAB IHC-stained slides of the nine MDOTSCC samples, including cells within the TNs and those within the peri-tumoral stroma, at 400× magnification. Fields of views were selected on regions that exhibited the highest density of staining. The proportion of cells within the TNs and the peri-tumoral stroma stained positively in each field of view was calculated using Excel (Microsoft Office, 2013), and results were subjected to *t*-tests for related samples, using SPSS v.22 statistical package. Total cell counts were also subjected to χ^2^ statistical analysis, comparing relative abundance of each cathepsin.

### Enzymatic Activity Assays

Enzyme activity assays for cathepsin B (cat# ab65300, Abcam), cathepsin D (cat# ab65302, Abcam), and cathepsin G (cat# ab126780, Abcam) were performed on three snap-frozen MDOTSCC samples of the cohort of six patients used for NanoString mRNA analysis, according to the manufacturer’s protocol. A snap-frozen tonsil sample was used as an appropriate positive control for the cathepsins B (17) and D (18) assays, and a denatured sample of the same tonsil was used as an appropriate negative control. The capthepsin G assay kit was supplied with its positive and negative controls. The results were obtained using the Variskan Flash plate reader (Thermo Fisher Scientific). Experiments were performed in duplicates with averages taken for each.

### Image Analysis

3,3-Diaminobenzidine IHC-stained and CISH-stained slides were viewed and imaged on Olympus BX53 light microscope (Olympus). IF IHC-stained slides were viewed and imaged using Olympus FV1200 confocal laser-scanning microscope and processed with cellSens Dimension 1.11 software using 2D deconvolution algorithm (Olympus).

## Results

### Histochemical and 3,3-DAB IHC Staining

**Figure 1:**
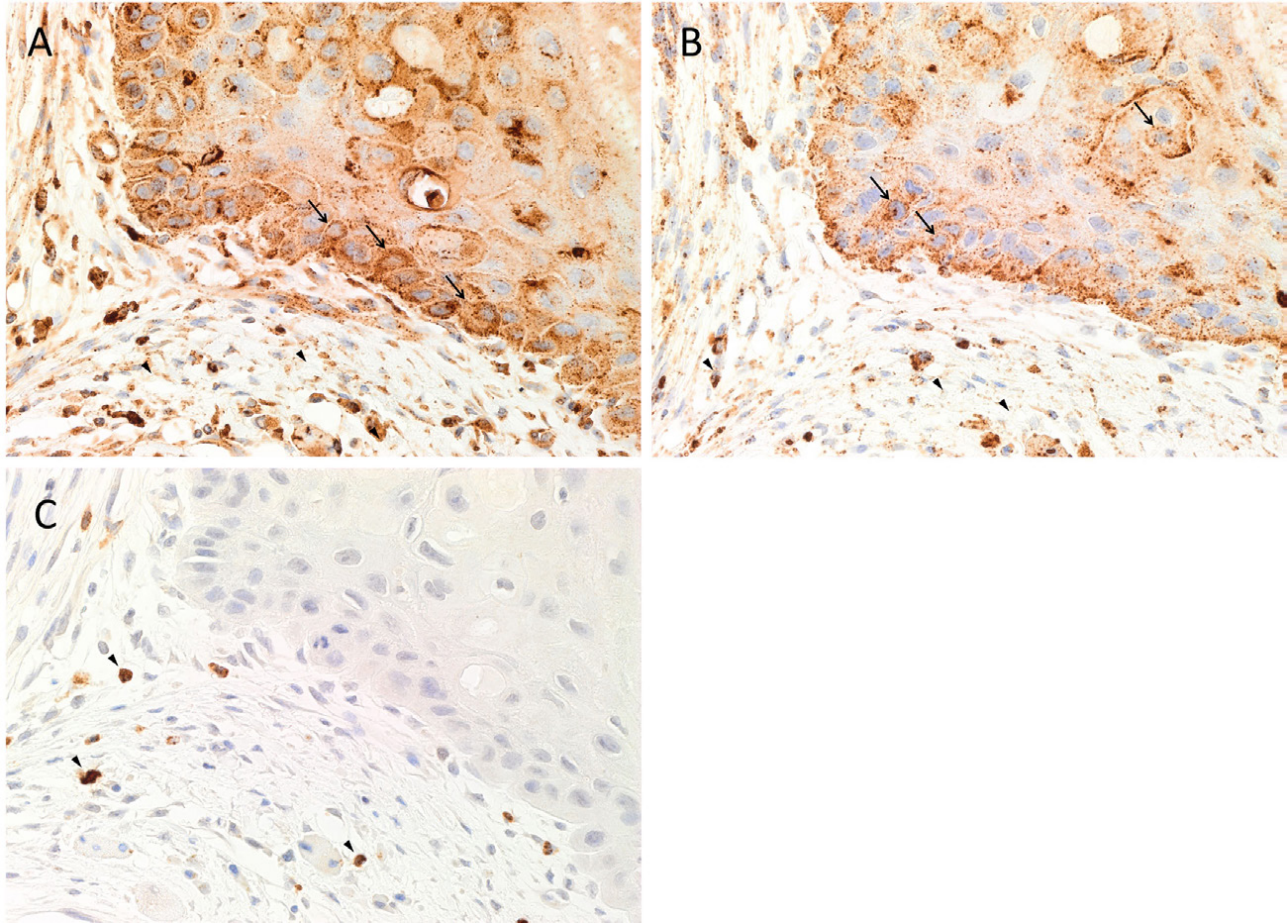
Representative 3,3-diaminobenzidine immunohistochemical-stained sections of moderately differentiated oral tongue squamous cell carcinoma demonstrating cytoplasmic expression of cathepsin B [**(A)**, brown] within the tumor nests (TNs) and the peri-tumoral stroma. Granular staining of cathepsin D [**(B)**, brown] was present predominantly on cells within the TNs and those within the peri-tumoral stroma. Cytoplasmic expression of cathepsin G [**(C)**, brown] was demonstrated in cells within the peri-tumoral stroma. Nuclei were counter-stained with hematoxylin [**(A–C)**, blue]. Original magnification: 400×.

Hematoxylin and eosin staining of 4 μm-thick, formalin-fixed, paraffin-embedded sections of all nine MDOTSCC samples confirm the presence and appropriate histological grading. 3,3-Diaminobenzidine IHC staining demonstrated cytoplasmic expression of cathepsin B (Figure 1A, brown) by cells predominantly within the TNs (Figure 1A, brown, *arrows*) and cells within the peri-tumoral stroma (Figure 1A, brown, *arrowheads*). Granular cytoplasmic staining of cathepsin D (Figure 1B, brown) was localized to cells within the TNs (Figure 1B, brown, *arrows*) and some cells within the peri-tumoral stroma (Figure 1B, brown, *arrowheads*). Cathepsin G was expressed in the cytoplasm of only some cells within the peri-tumoral stroma (Figure 1C, brown, *arrowheads*).

Positive controls for cathepsins B (Figure S1A in Supplementary Material, brown), D (Figure S1B in Supplementary Material, brown), and G (Figure S1C in Supplementary Material, brown) demonstrated expected staining patterns in human placenta (19) and breast cancer (20), and mouse bone marrow (21), respectively. The negative control showed minimal staining (Figure S1D in Supplementary Material, brown).

### IF IHC Staining

To localize cathepsins B, D, and G in relation to the CSC sub-populations, IF IHC staining was performed on two representative MDOTSCC samples from the original cohort of nine patients used for DAB IHC staining.

**Figure 2:**
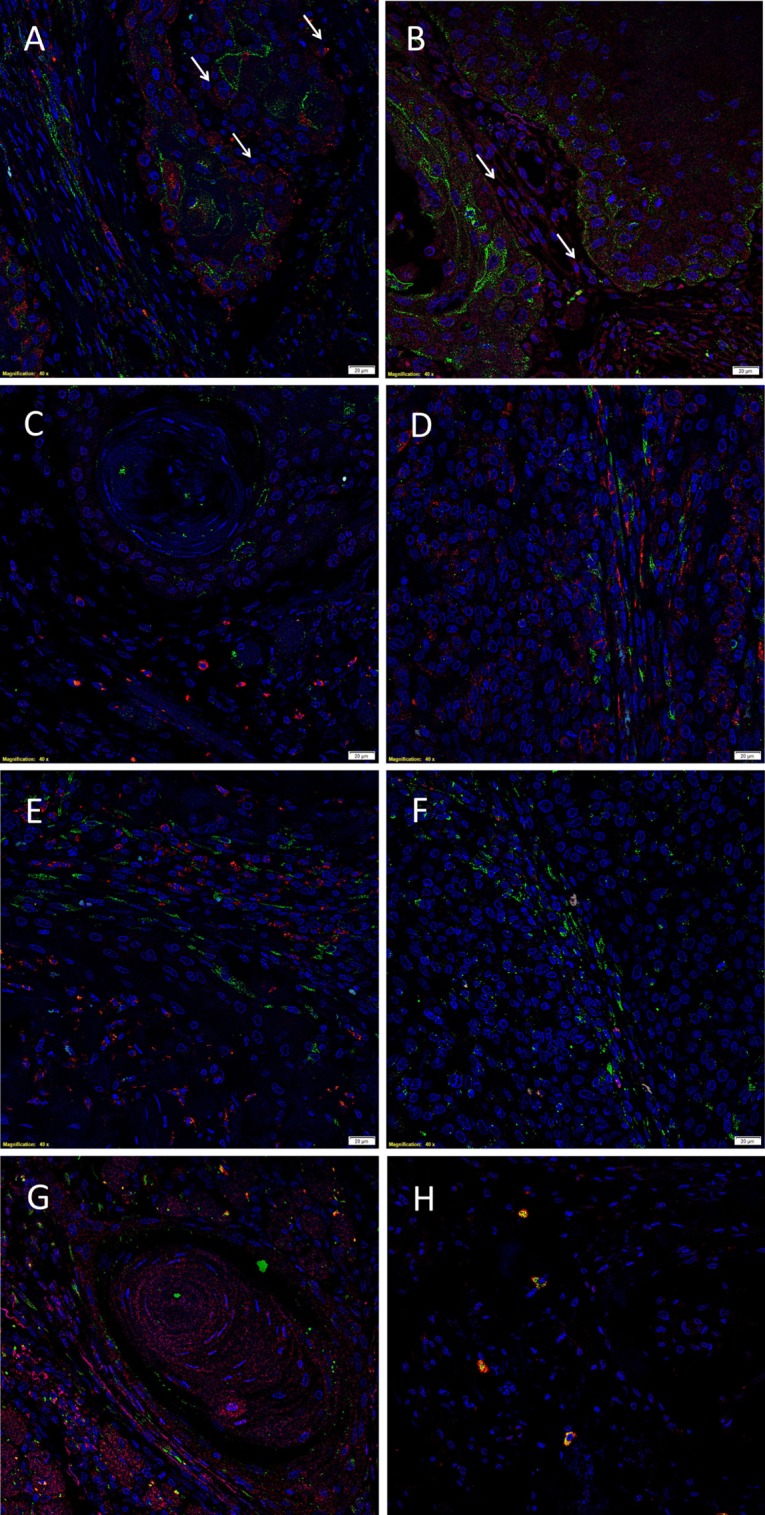
Representative immunofluorescent immunohistochemical-stained sections of moderately differentiated oral tongue squamous cell carcinoma demonstrating expression of cathepsin B [**(A,D)**, red] by the EMA^+^ [**(A)**, green] cells within the tumor nests (TNs), and the OCT4^+^ [**(D)**, green] cells within the peri-tumoral stroma. Cathepsin D [**(B,E)**, red] was expressed by the EMA^+^ cells within the TNs [**(B)**, green], but not the OCT4^+^ cells within the peri-tumoral stroma [**(E)**, green]. Cathepsin D [**(G)**, red] was not co-localized to the cells that expressed tryptase [**(G)**, green]. Cathepsin G [**(C,F)**, red] was not expressed by the EMA^+^ cells within the TNs [**(C)**, red] or the OCT4^+^ cells within the peri-tumoral stroma [**(F)**, red]. The tryptase^+^ cells [**(H)**, green] within the peri-tumoral stroma also expressed cathepsin G [**(H)**, red]. All slides were counter-stained with 4′,6′-diamino-2-phenylindole. Scale bars: 20 µm.

The EMA^+^ cells (Figures 2A–C, green) within the TNs (6) expressed both cathepsin B (Figure 2A, red) and cathepsin D (Figure 2B, red), with no expression of cathepsin G (Figure 2C, red). Consistent with the results of DAB IHC staining, IF IHC staining demonstrated immunoreactivity (IR) for cathepsin B (Figure 2A, red, *arrows*) and cathepsin D (Figure 2B, red, *arrows*) by cells within the peri-tumoral stroma. The CSC subpopulation within the peri-tumoral stroma of MDOTSCC which expresses OCT4 (6) (Figures 2D–F, green) demonstrated IR for cathepsin B (Figure 2D, red), but not cathepsin D (Figure 2E, red) or cathepsin G (Figure 2F, red).

To further characterize the cathepsin D^+^ (Figure 2G, red) and cathepsin G^+^ (Figure 2H, red) cells, we performed co-staining with tryptase, as we have reported in infantile hemangioma (14). This confirmed that tryptase (Figures 2G,H, green) was not expressed by the cathepsin D^+^ (Figure 2G, red) cells, but was expressed by the cathepsin G^+^ (Figure 2H, red) cells.

Images illustrating the individual stains demonstrated in Figure 2 are presented in Figure S2 in Supplementary Material. Minimal staining was present on the negative control (Figure S2Q in Supplementary Material), confirming the specificity of the primary antibodies used.

### NanoString mRNA Analysis

**Figure 3:**
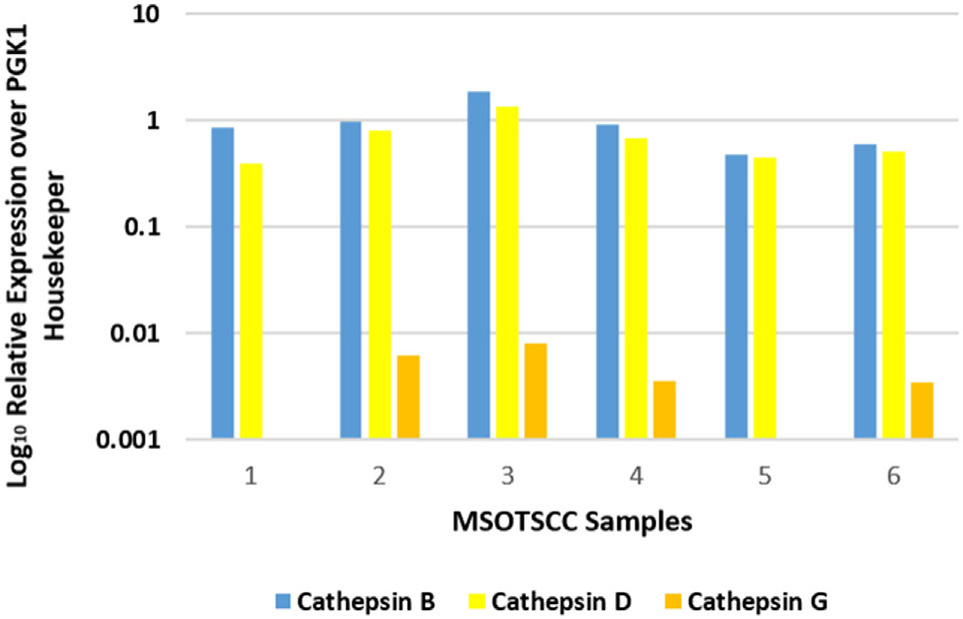
Relative expression of mRNA transcripts of cathepsins B, D, and G over the PGK1 housekeeper gene in six moderately differentiated oral tongue squamous cell carcinoma samples. Transcriptional profiling confirmed the presence of cathepsins B and D in all six samples. Cathepsin G was detectable in four of the six samples.

NanoString mRNA analysis for cathepsins B, D, and G was normalized against the housekeeping gene, PGK1, confirming transcriptional activation for both cathepsins B and D in all six MDOTSCC samples, whereas cathepsin G was detected at low levels in four of the six samples studied (Figure 3).

Statistical analysis of the gene transcripts confirmed significantly greater presence of cathepsin B than cathepsin D (*t* = 3.073, *p* < 0.05), and that both cathepsins B and D were significantly more abundant than cathepsin G (*t* = 4.701 and *t* = 4.885, respectively, *p* < 0.01).

### Colorimetric *In Situ* Hybridization

**Figure 4:**
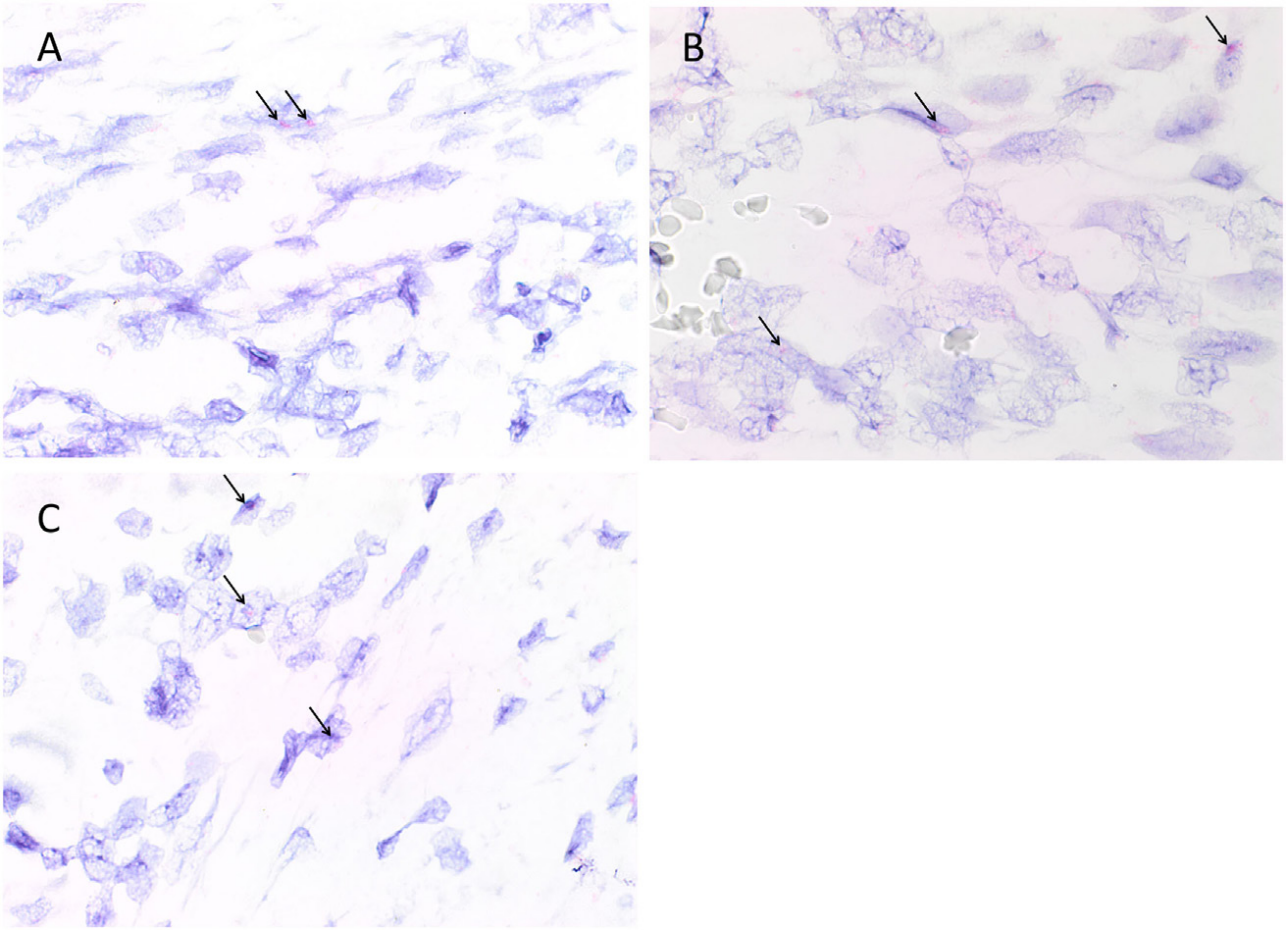
Representative colorimetric *in situ* hybridization stained sections of moderately differentiated oral tongue squamous cell carcinoma demonstrating mRNA expression of cathepsin B [**(A)**, pink], cathepsin D [**(B)**, pink], and cathepsin G [**(C)**, pink]. Original magnification: 1,000×.

Colorimetric *in situ* hybridization confirmed the presence of mRNA for cathepsin B (Figure 4A, pink, *arrows*), cathepsin D (Figure 4B, pink, *arrows*), and cathepsin G (Figure 4C, pink, *arrows*) in cells within all six MDOTSCC samples examined.

Appropriate staining was seen on positive controls for cathepsin B (Figure S3A in Supplementary Material, pink, *arrows*), cathepsin D (Figure S3B in Supplementary Material, pink, *arrows*), and cathepsin G (Figure S3C in Supplementary Material, pink, *arrows*), indicating the presence of mRNA transcripts in the controls. The negative control performed on each run showed the absence of mRNA transcripts for any marker (Figure S3D in Supplementary Material).

### Cell Counting and Statistical Analyses

**Figure 5:**
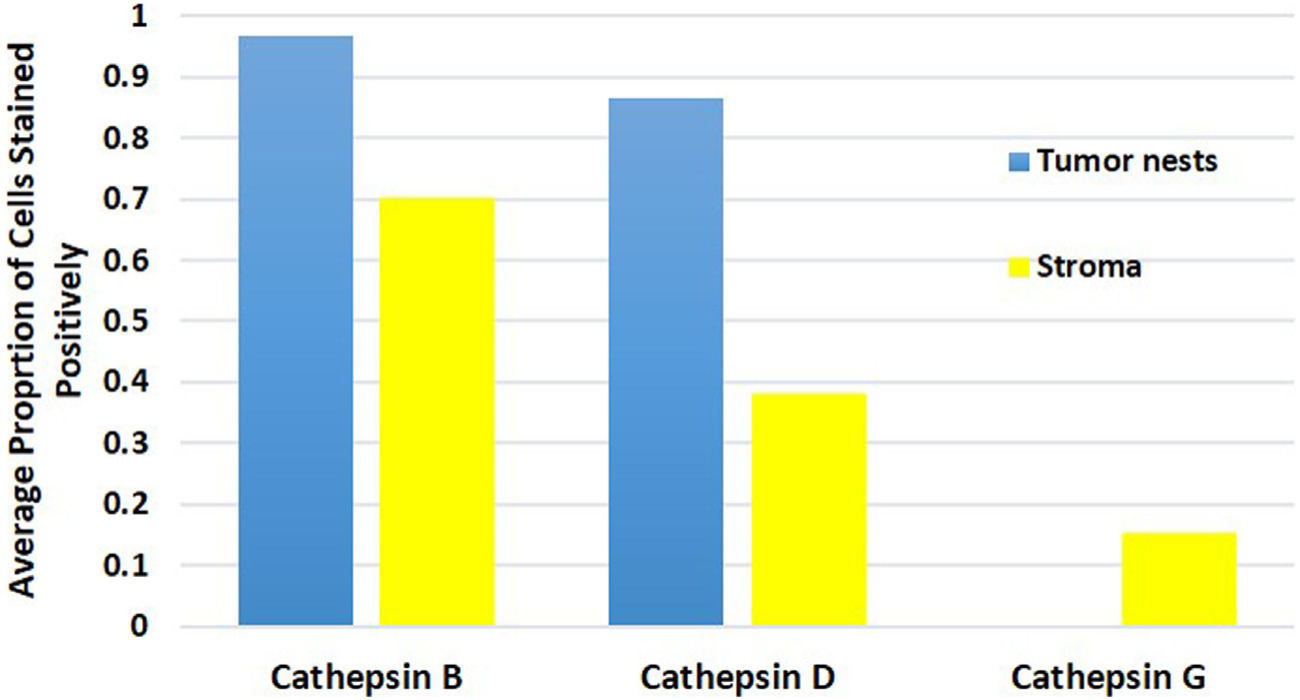
Average proportion of cells within the tumor nests and cells within the peri-tumoral stroma in moderately differentiated oral tongue squamous cell carcinoma stained positively for cathepsin B, cathepsin D, and cathepsin G.

Cell counting for cathepsins B, D, and G (Figure 5) in all nine samples demonstrated significantly more cells staining positively for cathepsin B within the TNs, than those within the peri-tumoral stroma (91% vs. 77%, *t* = 10.281, *p* < 0.000). Analysis using χ^2^ method showed that there were significantly more cathepsin B^+^ cells than cathepsin D^+^ cells (χ^2^ = 409.9.261, *p* < 0.0001), and that there was significantly more cathepsin D^+^ cells relative to the cathepsin G^+^ cells (χ^2^ = 3356.0, *p* < 0.0001). Consequently, we conclude that there was significantly more cathepsin B than cathepsin G.

### Enzymatic Activity Assays

**Figure 6:**
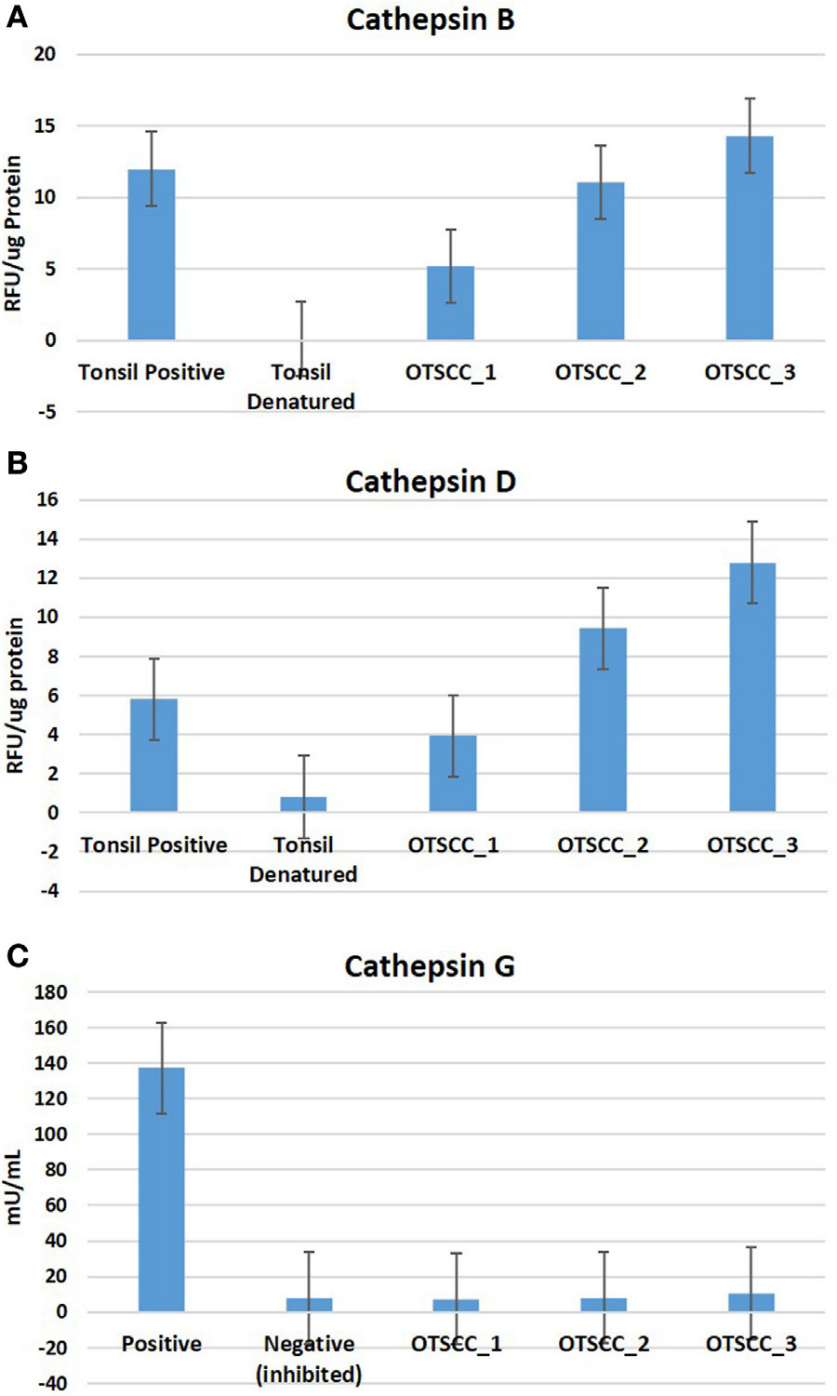
Averages for the enzymatic activity assays of three moderately differentiated oral tongue squamous cell carcinoma samples for cathepsin B **(A)**, cathepsin D, **(B)** and cathepsin G **(C)** with a tonsillar positive and negative (denatured) control tissues for cathepsins B and D; and the manufacturer’s controls for cathepsin G.

To determine the functionality of the cathepsins B, D, and G within MDOTSCC, we performed enzymatic activity assays, which confirmed the functional activity for cathepsin B (Figure 6A) and cathepsin D (Figure 6B), but not cathepsin G (Figure 6C) within all three MDOTSCC samples, as compared with the expected activity for the positive and negative controls (Figures 6A–C).

## Discussion

We have recently demonstrated expression of components of the RAS by the two CSC subpopulations within MDOTSCC (13). The novel finding of the expression and localization of cathepsins B, D, and G to these CSC subpopulations provides further insights into the biology of this cancer.

It is intriguing that cathepsin B is present in both CSC subpopulations within the TNs and the peri-tumoral stroma. However, cathepsin D is localized only to the CSC subpopulation within the TNs, while cathepsin G is localized exclusively to the tryptase^+^ phenotypic mast cells within the peri-tumoral stroma, similar to the finding in infantile hemangioma (14).

The expression of cathepsin B has been recently reported in oral cavity SCC (22). Yang et al. (22) have demonstrated that increased expression of cathepsin B is correlated with lymph node metastasis, higher tumor grade, and significantly poorer overall survival.

The finding that only four of the six MDOTSCC samples displayed relative low abundance of cathepsin G by NanoString mRNA analysis may be due to either rapid degradation of mRNA for this protein or sampling bias. However, the low transcriptional abundance may reflect the relatively low numbers cells that stained positively for cathepsin G by IHC staining.

Cathepsins B, D, and G exist in precursor forms, with the mature form of cathepsin B being 29 kDa, and its precursor form being 39 kDa (23). Its conversion requires a single cleavage of the precursor protease by either cathepsin B or in acidic conditions (23, 24). Cathepsin D has two precursor forms—the 51 kDa pre-pro-enzyme is cleaved, removing the signal peptide to form the 48 kDa pro-enzyme (25). Active cathepsin D is formed by removal of the 44 amino acid pro-domain of the pro-enzyme (25). Cathepsin D can be cleaved to form a single or two-chain form (26).

It is exciting to speculate that both cathepsin B and cathepsin D undergo “intra-tumoral” post-translational modification following synthesis, with the latter being confirmed by enzyme activity assays we have performed.

The precursor of cathepsin G is a 32.5 kDa protein which includes a signal peptide and a pro-dipeptide at the N terminus (27–29). This is cleaved by another protease, cathepsin C, to form the active 28.5 kDa cathepsin G (27, 29). Although we have confirmed transcriptional activation for cathepsin G and demonstrated localization of this protein to cells by IHC staining within the MDOTSCC samples, we used in this study, it was not possible to determine whether cathepsin G would undergo posttranslational modification into the active form, based on the activity data. However, given the relatively low number of cathepsin G^+^ cells, such activity may not be detectable by the activity kit used in this study. Furthermore, it is interesting that cathepsin G is expressed by mast cells, consistent with previous reports (14). However, its precise role in MDOTSCC carcinogenesis remains to be determined.

Recent literature suggests that the RAS plays a crucial role in cancer growth and metastasis (8, 9), specifically cell proliferation (10). We have demonstrated expression of cathepsins B and D by the two CSC subpopulations, and the expression of cathepsin G by the phenotypic mast cells within MDOTSCC that we have recently identified. This suggests the putative presence of bypass loops for the RAS in MDOTSCC (30).

This report suggests CSCs as a potential therapeutic target for MDOTSCC, through modulation of cathepsin B and D, and potentially G, in addition to modulation of the classical RAS. A larger study is needed to validate these findings.

### Limitations

1. Further study with a bigger sample size would be needed to confirm the observation of this study.
2. Further study including well and poorly differentiated OTSCC, in addition to moderately differentiated lesions may improve understanding of this aggressive cancer.
3. Functional study using *in vitro* and *in vivo* models of MDOTSCC would be needed to further validate the results of this study.

### Take Home Messages

1. Cathepsins B, D, and G are expressed by MDOTSCC.
2. Cathepsin B is localized to the CSC subpopulations within the TNs and the peri-tumoral stroma.
3. Cathepsin D is localized to the CSC subpopulation within the TNs.
4. Cathepsin G is localized to the phenotypic mast cells within the peri-tumoral stroma.
5. Cathepsins B and D are active in MDOTSCC.
6. These novel findings suggest CSCs within MDOTSCC as a potential therapeutic target by modulating the RAS.

## Ethics Statement

This study was approved Central Regional Health and Disability Ethics Committee (ref. no. 12/CEN/74).

## Author Contributions

TI and ST formulated the study hypothesis and designed the study. TF, HB, TI, and ST interpreted the IHC staining data. TF and TI interpreted the NanoString data. TF performed cell counting. BvS performed the enzymatic activity assays and interpreted the results. RM performed statistical analysis. TF, TI, and ST drafted the manuscript. All authors approved the manuscript.

## Conflict of Interest Statement

The authors declare that the research was conducted in the absence of any commercial or financial relationships that could be construed as a potential conflict of interest. TI and ST are inventors of the PCT patent application (no. PCT/NZ2015/050108) Cancer Diagnosis and Therapy.

## Supplementary Material

Figure S1 Representative 3,3-diaminobenzidine immunohistochemical stained sections of positive control tissues showing staining for cathepsin B in human placenta [**(A)**, brown], cathepsin D in human breast cancer [**(B)**, brown], and cathepsin G in mouse bone marrow [**(C)**, brown]. A moderately differentiated oral tongue squamous cell carcinoma sample was used as a negative control by using an IgG isotype control[**(D)**, brown].

Figure S2 Split immunofluorescent immunohistochemical-stained images demonstrated in Figure 2 for cathepsin B [**(A,G)**, red], cathepsin D [**(C,I,M)**, red], cathepsin G [**(E,K,O)**, red], EMA [**(B,D,F)**, green], OCT4 [**(H,J,L)**, green] and tryptase [**(N,P)**, green]. Cell nuclei were counterstained with 4′,6′-diamino-2-phenylindole [**(A–Q)**, blue]. A moderately differentiated oral tongue squamous cell carcinoma sample was used as a negative control **(Q)** by using primary isotype mouse and rabbit antibodies.

Figure S3 Representative colorimetric in situ hybridization stained sections of positive control tissues showing positive staining of cathepsin B in human placenta [**(A)**, pink], cathepsin D in human breast cancer [**(B)**, pink], and cathepsin G in mouse bone marrow [**(C)**, pink]. A moderately differentiated oral tongue squamous cell carcinoma samples was used as a negative control[**(D)**, pink] using a Bacillus probe.
