# Supplementary figures and images for: Expression and Localization of Cathepsins B, D, and G in Two Cancer Stem Cell Subpopulations in Moderately Differentiated Oral Tongue Squamous Cell Carcinoma
Source: Front Med (Lausanne). 2017 Jul 20;4:100. doi: 10.3389/fmed.2017.00100 (PMC5517773; doi:10.3389/fmed.2017.00100)

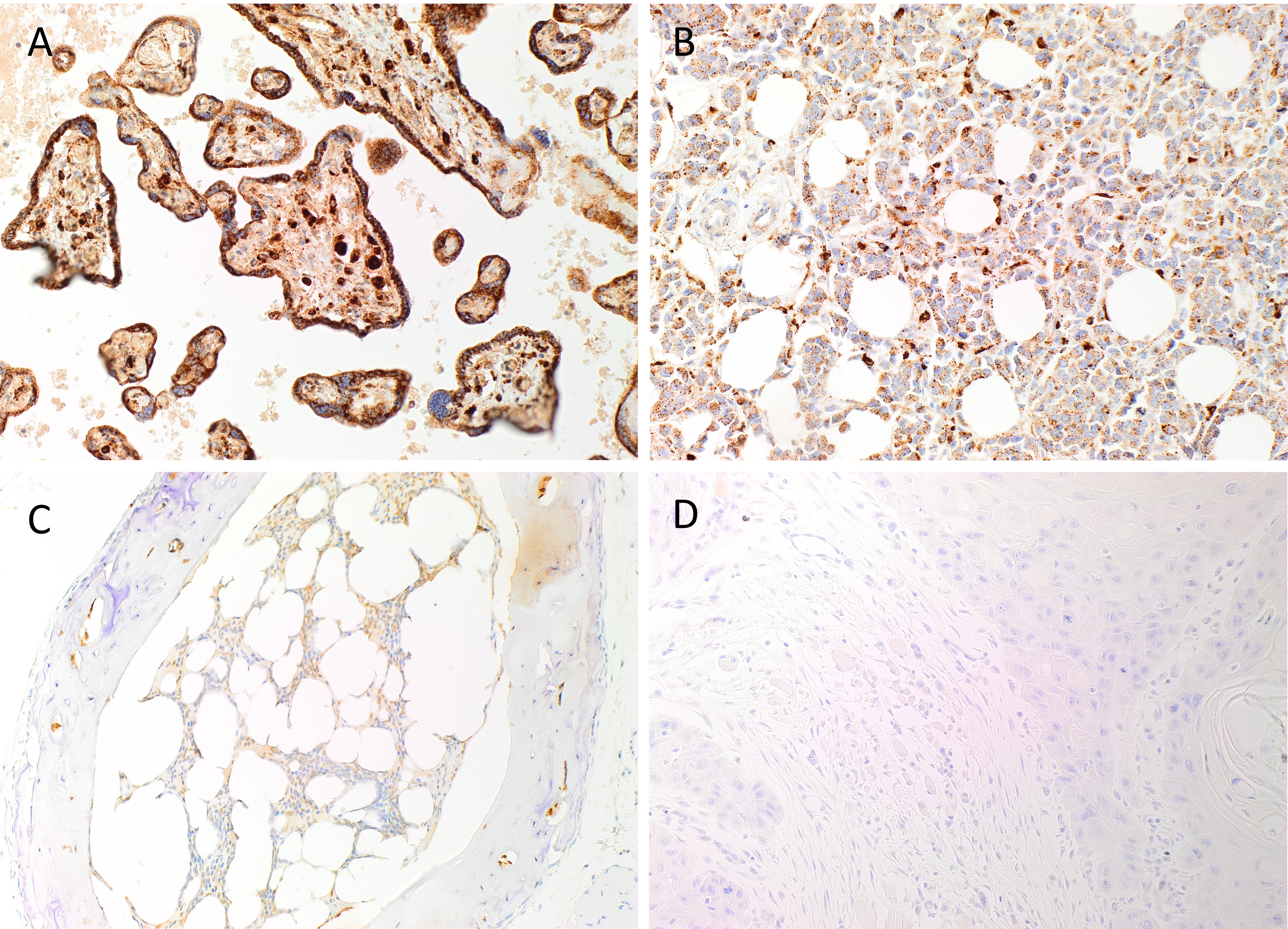

Supplement: Figure S1 — Representative 3,3-diaminobenzidine immunohistochemical stained sections of positive control tissues showing staining for cathepsin B in human placenta [(A), brown], cathepsin D in human breast cancer [(B), brown], and cathepsin G in mouse bone marrow [(C), brown]. A moderately differentiated oral tongue squamous cell carcinoma sample was used as a negative control by using an IgG isotype control[(D), brown]. [file Image_1.JPEG]

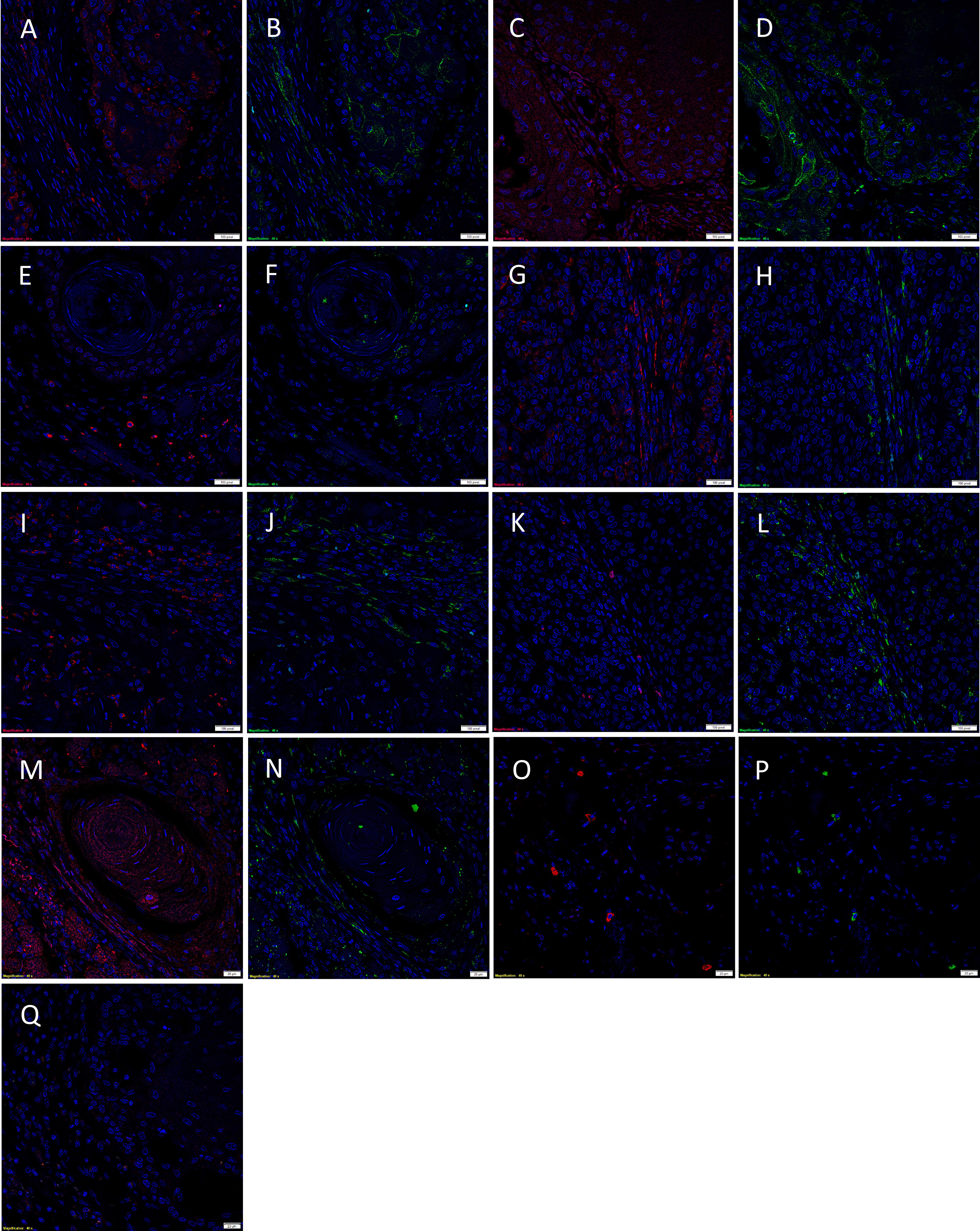

Supplement: Figure S2 — Split immunofluorescent immunohistochemical-stained images demonstrated in Figure 2 for cathepsin B [(A,G), red], cathepsin D [(C,I,M), red], cathepsin G [(E,K,O), red], EMA [(B,D,F), green], OCT4 [(H,J,L), green] and tryptase [(N,P), green]. Cell nuclei were counterstained with 4′,6′-diamino-2-phenylindole [(A–Q), blue]. A moderately differentiated oral tongue squamous cell carcinoma sample was used as a negative control (Q) by using primary isotype mouse and rabbit antibodies. [file Image_2.jpg]

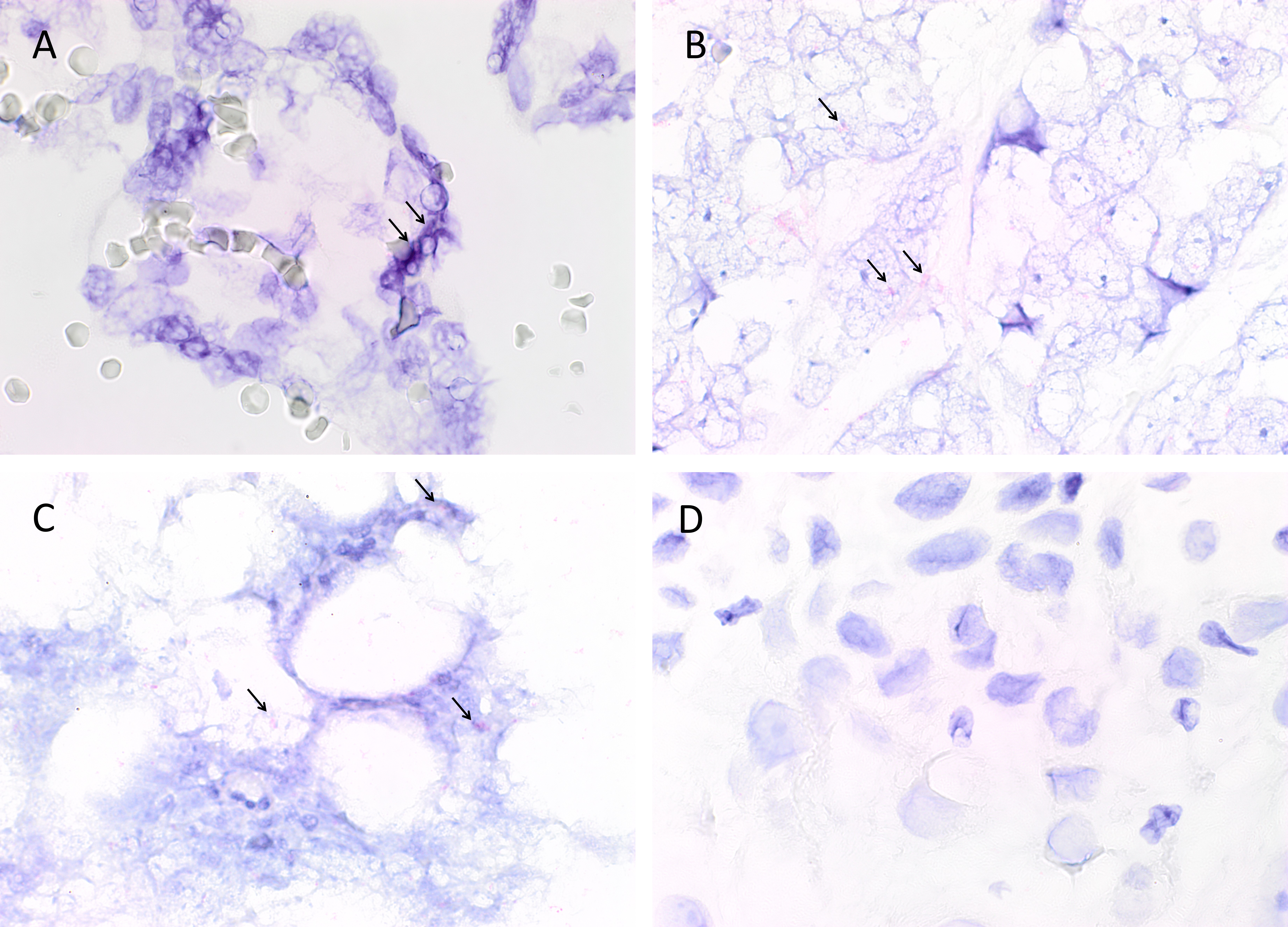

Supplement: Figure S3 — Representative colorimetric in situ hybridization stained sections of positive control tissues showing positive staining of cathepsin B in human placenta [(A), pink], cathepsin D in human breast cancer [(B), pink], and cathepsin G in mouse bone marrow [(C), pink]. A moderately differentiated oral tongue squamous cell carcinoma samples was used as a negative control[(D), pink] using a Bacillus probe. [file Image_3.jpeg]
